# Editorial: Advances in preventing suicide among veterans

**DOI:** 10.3389/fpsyt.2025.1689946

**Published:** 2025-09-03

**Authors:** Joseph Mignogna, Bryann DeBeer

**Affiliations:** ^1^ Rocky Mountain Mental Illness Research, Education, and Clinical Center (MIRECC) for Suicide Prevention, Rocky Mountain Regional VA Medical Center, Aurora, CO, United States; ^2^ Department of Physical Medicine and Rehabilitation, University of Colorado Anschutz Medical Campus, Aurora, CO, United States

**Keywords:** suicide prevention, suicide risk factors, rural veterans, neuroscience, lethal means safety, telehealth interventions, veterans

Preventing suicide among military veterans has been a high priority in the United States since the early 2000s. In 2022, suicide rates among veterans were 1.5 times greater than non-veterans. This rate has been lower for veterans in all but 2 of the past 14 years, with the exceptions being 2020 and 2021, which showed a slight increase. The collection of articles in this Research Topic showcases evidence of recent novel interventions and investigations advancing our understanding of preventing suicide among military veterans. These articles range from investigations illuminating suicide mechanisms through basic science and neuroscience, to research on interventions and an implementation trial, and studies focused on special populations at risk.

Basic science and neuroscience offer the opportunity to identify novel mechanisms that could be used as interventional targets. The neuroscience of suicide risk, and related potential interventions, has yet to be fully understood. McGlade et al. add to this literature through their investigation of the neurobiological underpinnings of attention speed. Results indicated that for participants who had made a prior suicide attempt, attention speed was slower than for participants who had only experienced suicidal ideation or had experienced neither suicidal ideation nor attempt. Overall, challenges with attention speed were associated with lower right rostral anterior cingulate cortex gray matter volume in those who had experienced a suicide attempt in comparison to those who had not experienced suicidal ideation or behavior. However, within this research, the team found substantial sex differences, highlighting the need for further investigation as well as the tailoring of suicide prevention to specific populations. Detecting risk for suicide has been a major challenge within the field. Myers et al. studied an innovative strategy for assessing for near-term risk of suicide attempts. This research demonstrated that individuals who had an upcoming actual suicide attempt interpreted ambiguous outcomes similarly to mild punishing events, while groups that had other suicidal behavior or suicidal ideation, and a group that experienced no suicide-related ideation or behavior, understood ambiguous events to be mildly rewarding. The reinforcement values then predicted actual suicide attempts. Reinforcement learning may be an important avenue for interventional development related to suicide prevention. Overall, these basic science findings offer innovative findings that afford the opportunity to propel the field forward.

Suicide prevention among rural veterans was the focus of the telehealth and peer-led intervention described in Goodman et al. case study. Relative to veterans residing in urban areas, those residing in rural areas have a 28.3% higher rate of suicide (1). Additionally, rural veterans face numerous stressors, including limited access to healthcare services (2), fewer employment opportunities, higher rates of chronic illness and disability (3), and social isolation (4). Importantly, these veterans are also more likely to own a firearm, a factor that increases suicide risk among veterans experiencing mental health and/or substance use problems (5). Goodman et al. describe their use of Project Life Force, a telehealth group intervention that uses Dialectical Behavior Therapy skills to augment suicide safety planning and lethal means safety counseling to assist veterans at risk for suicide. In seeking to enhance the success of veterans engaging with and benefiting from this intervention, fellow veterans with lived experience in firearm ownership and suicide behaviors (i.e., peer recovery leaders) were integrated into this 10-session treatment. This slight adaptation of including a peer recovery leader to encourage veterans participating in treatment was well-received and thought to be a key factor in the success of the Project Life Force group.

Given the evidence-practice gap that exists for the real-world use of evidence-based suicide prevention interventions (6), implementation-focused studies like Decker et al. formative evaluation of Dialectical Behavior Therapy Skills Groups across four VHA sites are needed. Similarly, with goals of increasing implementation of suicide prevention best practices into real-world settings, Landes et al. pilot tested the feasibility of mailing lethal means safety devices to veterans calling the Veterans Crises Line. In addition to soliciting important feedback from VCL responders about their comfort and experiences with incorporating mailing devices into their practice, relevant data were also captured that speak directly to the feasibility of a program like this, for example, data on the number of gun locks or medication takeback envelopes, if the length of call time increased, etc. These data enable subsequent implementation efforts to better prepare, allowing them to more fully anticipate what to expect.

Focusing and tailoring suicide prevention efforts is of critical importance when considering veteran subpopulations that are even at higher risk. Asian American, Native Hawaiian, and Pacific Islander (AANHPI) veterans are one such group requiring tailored suicide prevention efforts. Between 2001 and 2022, AANHPI veterans experienced an approximately 190% increase in suicide rate (1). Krishnamurti et al. qualitative study of Asian American veterans with a history of suicidal experiences explored these veterans’ identities, values, experiences in the military, as well as their experiences and perspectives related to suicidal thoughts and behaviors. Stigma surrounding mental health and help-seeking among Asian American veterans, along with experiencing the “model-minority” stereotypes, can result in notable barriers to receiving timely mental health and suicide prevention services.

Also highlighting efforts or prevent suicide among AANHPI veterans, Sells et al. mixed methods study sought to better understand factors increasing suicide risks among veterans residing in Guam, a United States island territory in the Pacific that is geographically closer to Japan than it is to the continental United States. Also explored were barriers, facilitators, and the utility of implementing a community-based suicide prevention program for rural veterans, namely Together with Veterans (TWV). This investigation identifies how veterans face unique suicide risks associated with being geographically isolated, experiencing conflicts in their cultural identity, stigma, and substantial obstacles to accessing mental health services. At the same time, veterans were viewed as resilient, and community members were optimistic about their capacity to achieve goals in reducing suicide. Community-based interventions like Together With Veterans are vital to efforts aiming to make a meaningful impact in preventing suicide, as they provide a culturally tailored approach that meets veterans where they live, fostering the opportunity for collaboration and coordination across organizations in and outside of healthcare in an effort to bring about a meaningful reduction in suicide prevention.

Overall, the collection of research presented in this Research Topic represents novel investigations and innovations focused on veteran suicide prevention. This research spans the gamut of work from basic science to intervention and implementation to focusing on tailoring prevention efforts to special populations at risk. Basic science and neuroscience findings provide the opportunity to understand basic mechanisms in suicide risk that can be used to inform the development of novel interventions. Intervention and implementation research provides an evidence base for specific interventional strategies to reduce the rate of death by suicide. Research on special populations, along with revelations of sex-based findings, emphasizes the importance of understanding specific risk factors in unique populations and tailoring interventions where needed. Ultimately, the collective goal of this research is to drive novel innovations to prevent veteran suicide.
